# A Broad m6A Modification Landscape in Inflammatory Bowel Disease

**DOI:** 10.3389/fcell.2021.782636

**Published:** 2022-01-19

**Authors:** Kai Nie, Jun Yi, Yuanyuan Yang, Minzi Deng, Yan Yang, Tianyu Wang, Xuejie Chen, Zhaoyu Zhang, Xiaoyan Wang

**Affiliations:** ^1^ Department of Gastroenterology, The Third Xiangya Hospital of Central South University, Changsha, China; ^2^ Hunan Key Laboratory of Nonresolving Inflammation and Cancer, Cancer Research Institute, Central South University, Changsha, China; ^3^ Department of Gastroenterology, The Xiangya Hospital of Central South University, Changsha, China; ^4^ Cancer Research Institute, Central South University, Changsha, China

**Keywords:** N6-methyladenosine, inflammatory bowel diseases, RNA modifications, epigenetics, biologics response

## Abstract

**Background and Aims:** N6-Methyladenosine (m6A) is the most common post-transcriptional modification on eukaryotic mRNA, affecting the mRNA’s fate. The role of m6A regulation in inflammatory bowel disease is unclear. Here, we investigated the m6A landscape in inflammatory bowel diseases (IBD).

**Methods:** Eleven human IBD microarray datasets were recruited from the Gene Expression Omnibus database and four were selected as discovery cohorts. An RNA-seq dataset from the Inflammatory Bowel Disease Multi’omics Database was used as a validation cohort. m6A regulators were measured in volunteers’ colonic samples. Consensus clustering and immune scoring were used to estimate the characteristics of m6A regulation in IBD. m6A-related characteristics of different sub-phenotypes, sample sources, and biological therapeutic responses were determined using seven independent datasets.

**Results:** m6A modification involves methyltransferases (writers), demethylases (erasers), and methylation-reading proteins (readers). A wide interaction exists between m6A regulators and IBD risk genes. The IBD risk loci can also be modified by m6A modifications in the public m6A sequencing data. Furthermore, m6A regulators displayed extensive differential expression in four independent discovery cohorts that share common differential genes (IGF2BP2, HNRNPA2B1, ZCCHC4, and EIF3I). In the validated cohort and enrolled volunteers’ colonic biopsy samples, the differential m6A regulators were reconfirmed. Two clusters of consensus clustering exhibit different immune phenotypes. m6A-modified positions exist in the core IBD immune cytokines. Another set of IBD datasets revealed m6A-related differences across clinical phenotypes, biological samples, and therapeutic response subgroups in IBD patients.

**Conclusion:** Regulation of m6A methylation is widely involved in IBD occurrence and development. m6A modifications in risk variants, core cytokines, immune cells, and other proteins may deeply influence the pathophysiology and clinical phenotypes. Further studies are needed to determine its role in IBD.

## Introduction

N6-Methyladenosine (m6A) in mRNAs was first discovered in the 1970s (Desrosiers et al., 1974) and later implicated in mRNA instability. Being the most extensive and frequent mRNA modification, m6A modification has emerged as a major research topic in epitranscriptomics (Zaccara et al., 2019; Huang et al., 2020). m6A landscapes in humans and mice were not described until the development of m6A-seq (also known as methylated RNA immunoprecipitation with next-generation sequencing, MeRIP-Seq) in 2012 (Dominissini et al., 2012; Meyer et al., 2012). m6A RNA methylation influences all stages of RNA processing, including precursor mRNA (pre-mRNA) splicing and primary microRNA (pri-miRNA) processing, nuclear export, translation, and degradation (Liu L. et al., 2020; Han et al., 2020). m6A modifications affect physiology at, at least three levels: 1) organismal or tissue level, influencing various biological processes, including development, infertility, and carcinogenesis), 2) cell signaling pathway level, including p53 and Notch signaling, and 3) at the machinery level, including spliceosome and the nuclear export machinery (Fu et al., 2014). Writers, erasers, and readers are enzymes that add, remove, or preferentially bind to the chemical modifications at designated m6A nucleotides. These functional components constitute a complex post-transcriptional system of gene regulation (Zaccara et al., 2019). m6A has been implicated in various pathologies, including cancer, inflammation, autoimmune diseases, and infections (Li et al., 2017; Han et al., 2020). However, the role of m6A modifications in inflammatory bowel diseases (IBD) is poorly understood.

IBDs are chronic intestinal disorders that typically fall into two subtypes: Crohn’s disease (CD) and ulcerative colitis (UC). Over 1 million residents in America and 2.5 million in Europe are estimated to have IBD, with substantial costs for health care (Kaplan, 2015). Moreover, IBD has emerged in newly industrialized countries in Asia, South America, and the Middle East and has evolved into a global disease with a rising prevalence in every continent (Kaplan, 2015). Several comorbid conditions have been proposed to be related to IBD, including cardiovascular disease, neuropsychological disorders, and metabolic syndrome with heavy disease burden (Argollo et al., 2019). UC is limited to the colon, with superficial mucosal inflammation that extends proximally in a contiguous manner and may cause ulcerations, severe bleeding, toxic megacolon, and fulminant colitis (Ungaro et al., 2017). In contrast, CD can affect any part of the digestive tract, often in a non-contiguous manner, and is characterized by transmural inflammation, which may cause complications such as fibrotic strictures, fistulas, and abscesses (Roda et al., 2020). It has been reported with respect to the potentially important pathophysiological signatures of UC and CD, such as differentially enriched immune-cell sub-populations and genetic variants (e.g., Nucleotide-binding oligomerization domain 2, NOD2). However, the mechanisms underlying IBD are not fully understood. Genome-wide association studies (GWASs) have identified risk variants, including NOD2, autophagy-related 16-like 1, interleukin 23 receptor, and interleukin 10 (Chang, 2020), but they are not clearly explained or mapped, and the genes commonly used to describe them are only putative. Moreover, in most cases, the biological functions of their products and interactions need delineation. Further understanding is required to determine the mechanism of specific variants affecting mRNA levels and consequently protein levels, so as to provide further insight into the mechanisms of IBD pathogenesis. However, many IBD variants may represent m6A modification loci that exert effects on gene expression. Immune dysfunction also influences IBD pathophysiological processes (Mitsialis et al., 2020). B-cells, dendrite cells (DCs), and T-cells are significantly involved in IBD, and strong evidence indicates global m6A modifications in innate and adaptive immune systems (Shulman and Stern-Ginossar, 2020). Several reports have linked m6A epigenetic modification to IBD indirectly. Studies support a single m6A regulator participating in the immune-associated colitis-like methyltransferase 14 (METTL14) deletion in T-cells trigger spontaneous colitis (Lu et al., 2020), and m6A reader ELAV-like RNA binding protein 1 (ELAVL1, also known as HuR) maintain colonic epithelial Paneth cells’ function (Xiao et al., 2019; Xu et al., 2021; Zhang et al., 2021). However, m6A, as the major mRNA modification, and its systemic modifying landscape in IBD have not yet been described. The unknown truth is m6A regulators’ network’s role in IBD. There is a need to uncover the m6A’s role in the pathogenesis, pathophysiology, clinical diagnosis, and treatment application of IBD. An important description of the missing link between IBD and m6A will bring new insights and directions for future studies. Here, we analyzed large-scale multi-IBD microarray and RNA-seq datasets to comprehensively describe the broad m6A modification landscape in IBD.

## Methods

### Data Screening

To investigate the m6A landscape in IBD patients, data was retrieved from the gene expression omnibus (GEO) using the key words “(Inflammatory bowel disease OR IBD) AND microarray expression data AND Homo sapiens.” Inclusion criteria: 1. IBD OR Crohn OR colitis; 2, Homo sapiens; 3, Expression profiling by array OR high throughput sequencing; 4, Sample size>50; 5, Submitting date<2020.10.31; 6, DataSets OR Series. Exclusion criteria: 1, Not coding-gene expression data; 2, Non-integral data with nonIBD samples (except for healthy controls); 3, Lack of healthy controls; 4, Nonstandard therapy; 5, Data not available or low data quality (i.e., no comparable subgroups or insufficient significant differential genes). For discovery cohorts, we also consider factors to uncover reliable findings, such as: 1, all colon samples; 2, shared platform and data-style; and, adult patients. We recruited cohorts with necessary information such as sample sources, patient group, disease state, and biologics’ therapy response for subgroups’ analysis. Detailed data processing and analysis flows were displayed in Supplementary Figure S1A.

### Association Analysis Between IBD Risk Loci and m6A Regulation

Over 240 Single Nucleotide Polymorphisms (SNPs) have been identified as risk loci for IBD (de Lange et al., 2017), and genes at these risk loci have been collected and sorted out (Jostins et al., 2012; Liu et al., 2015; Huang et al., 2017; Park and Jeen, 2019). m6A regulatory genes, including writers, erasers, and readers, were collected by literature review. IBD risk genes and m6A regulatory genes were analyzed using the STRING database (https://string-db.org) and an interaction network developed (Szklarczyk et al., 2019). The interaction map of IBD risk genes and m6A regulatory genes based on the network was visualized using Cytoscape (version: 3.7.1) (Shannon et al., 2003). The RMVar database (a database of functional variants involved in RNA modification, http://rmvar.renlab.org) (Wang et al., 2020; Luo et al., 2021) was searched to identify m6A-regulated IBD-associated risk loci. Next, m6A regulatory genes closely related to IBD were obtained based on published data of m6A-label-seq, m6A individual-nucleotide-resolution cross-linking and immunoprecipitation seq (miCLIP-seq), and droplet-assisted RNA targeting by single-cell sequencing (DART-seq). These m6A methylation risk variants, SNP locations, transcriptome regulation, and risk genes were visualized on a Circos diagram (Gu et al., 2014).

### The IBD m6A Signature in Discovery and Validated Cohorts

The GEO datasets GSE10616, GSE73661, GSE75214, and GSE126124 were used as discovery cohorts for analyzing differential m6A regulatory genes. All datasets were analyzed using the GEO2R online tool based on the R limma package (Ritchie et al., 2015). Overlapping gene expression profiles associated with m6A were obtained using Conway’s UpSetR R package (Conway et al., 2017). Correlation analysis between the m6A gene group was done on the Inflammatory Bowel Disease Multi’omics Database (IBDMDB, http://ibdmdb.org) (Lloyd-Price et al., 2019) using the stats R package on Sangerbox tools (http://www.sangerbox.com/tool). A heatmap of m6A gene expression in IBD was validated using a validation cohort from IBDMDB. For example, validation cohort IBDMDB is part of the American National Institute of Health’s integrative human microbiome project (HMP2/iHMP) (Lloyd-Price et al., 2019).

### Volunteer Recruitment, Reverse Transcription-Quantitative Polymerase Chain Reaction Measure

From June to November 2021, we collected IBD patients’ and healthy controls’ colonic biopsy samples under coloscopy. These samples are from the cohort in research on Key Technologies of Comprehensive Prevention and Treatment of IBD in Hunan Province, China. The Ethics Institutional Review Board of China’s Third Xiangya Hospital has approved this project’s cohort. A total of twelve volunteers were enrolled, and their information is listed in Table 1. At least two biopsy samples were collected for each volunteer. Total RNA from colon tissue was isolated by using TRIzol® (Thermo Fisher Scientific, Inc.). The qPCR measure was conducted as previously described (Luo et al., 2019) (primer sequences are presented in Table 2).

**TABLE 1:** Volunteers recruited in the experimental validation.

ID	Age	Gender	Diagnosis	Biopsy position	Sampling date
1	28	Male	Healthy control	Colon	16.11.2021
2	46	Male	Healthy control	Colon	17.11.2021
3	32	Male	Healthy control	Colon	16.11.2021
4	25	Male	Healthy control	Colon	16.11.2021
5	29	Female	Healthy control	Colon	16.11.2021
C1	33	Male	Crohn’s disease	Colon	6.9.2021
C2	16	Male	Crohn’s disease	Colon	27.8.2021
C3	26	Male	Crohn’s disease	Colon	18.8.2021
U1	52	Male	Ulcerative colitis	Colon	12.7.2021
U2	37	Female	Ulcerative colitis	Colon	25.6.2021
U3	39	Male	Ulcerative colitis	Colon	4.7.2021
U4	34	Male	Ulcerative colitis	Colon	27.6.2021

**TABLE 2:** The primer sequences.

Gene	Forward	Reverse
IGF2BP2	TGT​TGG​TGC​CAT​CAT​CGG​A	TTC​GGC​TAG​TTT​GGT​CTC​ATC​T
HNRNPA2B1	AAG​AGG​AGG​ATA​TGG​TGG​TGG​AG	GGA​CCG​TAG​TTA​GAA​GGT​TGC​TG
ZCCHC4	TCC​GTT​TGG​TGG​CTT​GGT​T	GGG​AAA​ATC​CAG​AAA​ATG​GGT​AG
GAPDH	GGA​AGC​TTG​TCA​TCA​ATG​GAA​ATC	TGA​TGA​CCC​TTT​TGG​CTC​CC

### Consensus Clustering in the IBD m6A Signature, Principal Component Analysis, Immune Scoring, and Clinical Correlation

Consensus clustering was performed to better distinguish the detailed m6A signature in IBDMDB subgroups using the consensus ClusterPlus R package (Swift et al., 2004). Heatmap and PCA were used to confirm m6A distinction between different m6A clusters in IBD. Furthermore, immune scoring results of 22 immune cells of different m6A clusters were analyzed using Xcell (https://xcell.ucsf.edu), an online tool that can be used for cell type enrichment analysis based on gene expression data from various immune cell types (Aran et al., 2017). C-reactive protein and erythrocyte sedimentation rate’s data of different samples were obtained from IBDMDB and analyzed using a non-paired t-test.

### The m6A Signature in IBDMDB

The IBDMDB validation cohort was used to analyze the correlation between IBD core cytokines and m6A regulatory genes using the stats R package (Team, 2013). Data on m6A methylation sites on IBD core cytokines were obtained from m6A-Atlas (www.xjtlu.edu.cn/biologicalsciences/atlas) (Tang et al., 2021). A detailed m6A expression profile was displayed for IBD and healthy controls in IBDMDB.

### A Global Landscape of Different Phenotypes of IBD Patients

Based on previous search results, GSE75214 was introduced for analysis of differential m6A gene expression in UC vs. CD, inflamed (active tissues) vs. uninflamed (inactive tissues) biopsies, and adult IBD vs. healthy controls. Dataset GSE6989 was used for comparison between pediatric IBD and healthy adult controls, GSE119600 for comparison between IBD whole blood RNA and healthy controls, GSE33943 for comparison between IBD peripheral blood leukocytes (PBLs) and healthy controls, and GSE3365 for comparison between IBD peripheral blood mononuclear cells (PBMCs) and healthy controls. These differential analyses were done using GEO2R based on the limma R package. Another three datasets were analyzed for the differential m6A signature between different therapeutic response subgroups (i.e., GSE73661 for infliximab and vedolizumab, GSE92415 for golimumab, and GSE112366 for ustekinumab).

## Results

### Data Searching and Processing

Finally, 11 datasets (GSE10616, GSE73661, GSE75214, GSE126124, GSE111889, GSE6989, GSE119600, GSE33943, GSE3365, GSE92415, and GSE112366) were screened out. Except for the microarray datasets, we also recruited a validatory RNA-seq dataset from IBDMDB (GSE111889) **(** Table 3). Of these datasets, GSE10616, GSE73661, GSE75214, and GSE126124 were analyzed as discovery cohorts, while GSE111889 was used as a validation cohort. GSE6989, GSE119600, GSE33943, GSE3365, GSE92415, and GSE112366 were further used to analyze the overall m6A landscape in IBD patients with various phenotypes (Table 4). To investigate the m6A’s role in IBD, we further explored the interactions of m6A regulators by database and IBD data, aiming to describe the m6A’s role in the pathogenesis, pathophysiology, and clinical outcome of IBD.

**TABLE 3:** The brief descriptions of the inclusive data series.

ID	Platform	Data	Sample	UC	CD	Control	Cohort
GSE10616	GPL5760	Microarray	Biopsy	10	32	11	Discovery
GSE73661	GPL6244	Microarray	Biopsy	167	0	11	Discovery
GSE75214	GPL6244	Microarray	Biopsy	74	75	22	Discovery
GSE126124	GPL6244	Microarray	Biopsy	20	39	39	Discovery
IBDMDB (GSE111889)	GPL11154	RNA-seq	Biopsy	73	86	49	Validation
GSE6989	GPL5760	Microarray	Biopsy	5	20	8	Pediatrics
GSE119600	GPL10558	Microarray	Blood	93	95	47	Blood
GSE33943	GPL570	Microarray	Blood	45 Unclassified IBDs		13	PBL
GSE3365	GPL96	Microarray	Blood	26	59	42	PBMC

**TABLE 4:** GEO datasets involved in IBD’s biologics response and m6A.

ID	Platform	Data	Sample	Biologics	Responder	Unresponder
GSE73661	GPL6244	Microarray	Biopsy	Infliximab	8	15
GSE73661	GPL6244	Microarray	Biopsy	Vedolizumab	22	37
GSE92415	GPL13158	Microarray	Biopsy	Golimumab	61	48
GSE112366	GPL13158	Microarray	Biopsy	Ustekinumab	132	113

### An Overview of m6A Regulation

**FIGURE 1:**
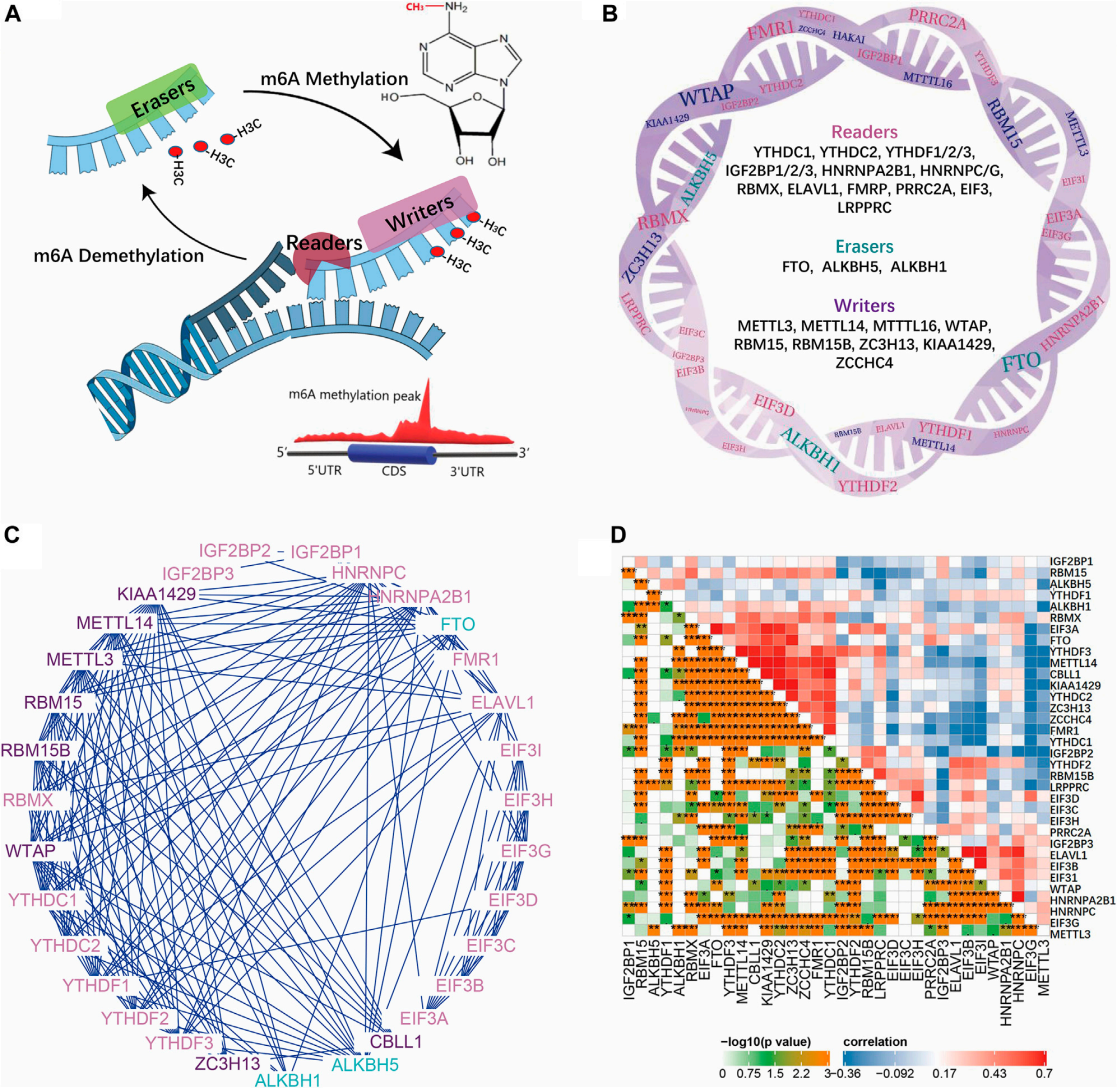
Mechanism of m6A modifications and the interaction of regulating genes. **(A)** is the reversible process of methylation on adenine which is modified by reader, writer and eraser proteins. **(B)** is the detailed gene list of reader, writer and eraser proteins. **(C)** is the interaction network of reader, writer and eraser proteins. **(D)** is the correlation map of m6A reader, writer and eraser proteins in IBDMDB cohort.

We first confirmed the global interactions of m6A regulators in the gene function database and IBD data, revealing their close functional gene set’s role. To better understand the m6A’s role in IBD, we should first get impressions about m6A regulators’ working mode and network. m6A modulators included writers (METTL3, METTL14, and METTL16), WTAP, RBM15, RBM15B, ZC3H13, KIAA1429, and ZCCHC4), erasers (FTO, ALKBH5, and ALKBH1), and readers (YTHDC1, YTHDC2, YTHDF1/2/3, IGF2BP1/2/3, HNRNPA2B1, HNRNPC/G, RBMX, ELAVL1, FMRP, PRRC2A, EIF3, and LRPPRC) (Figure 1B). Writers occur in a multicomponent m6A methyltransferase complex (MTC). The core MTC is comprised of METTL3 and METTL14. After deposition, the m6A methyl group can be removed by RNA demethylases (m6A erasers). m6A influences RNA fate by recruiting different m6A-binding proteins (m6A readers). MeRIP-seq relies on an antibody against m6A to immunoprecipitate fragmented RNA for subsequent deep sequencing (Meyer et al., 2012). Thus, m6A methylation peaks can be detected near the region between the coding DNA sequence (CDS) and the 3′ UTR, which is a common m6A methylation position that influences mRNA fate (Figure 1A). An interaction network between these m6A genes revealed a close relationship and mutual effect (Figure 1C). Further correlation analysis in IBDMDB revealed universal correlation between these m6A genes in IBD, and different m6A complexes exhibited clustering in the map (Figure 1D). These data indicate a close interaction between m6A genes.

### The Emerging m6A Landscape in IBD

**FIGURE 2:**
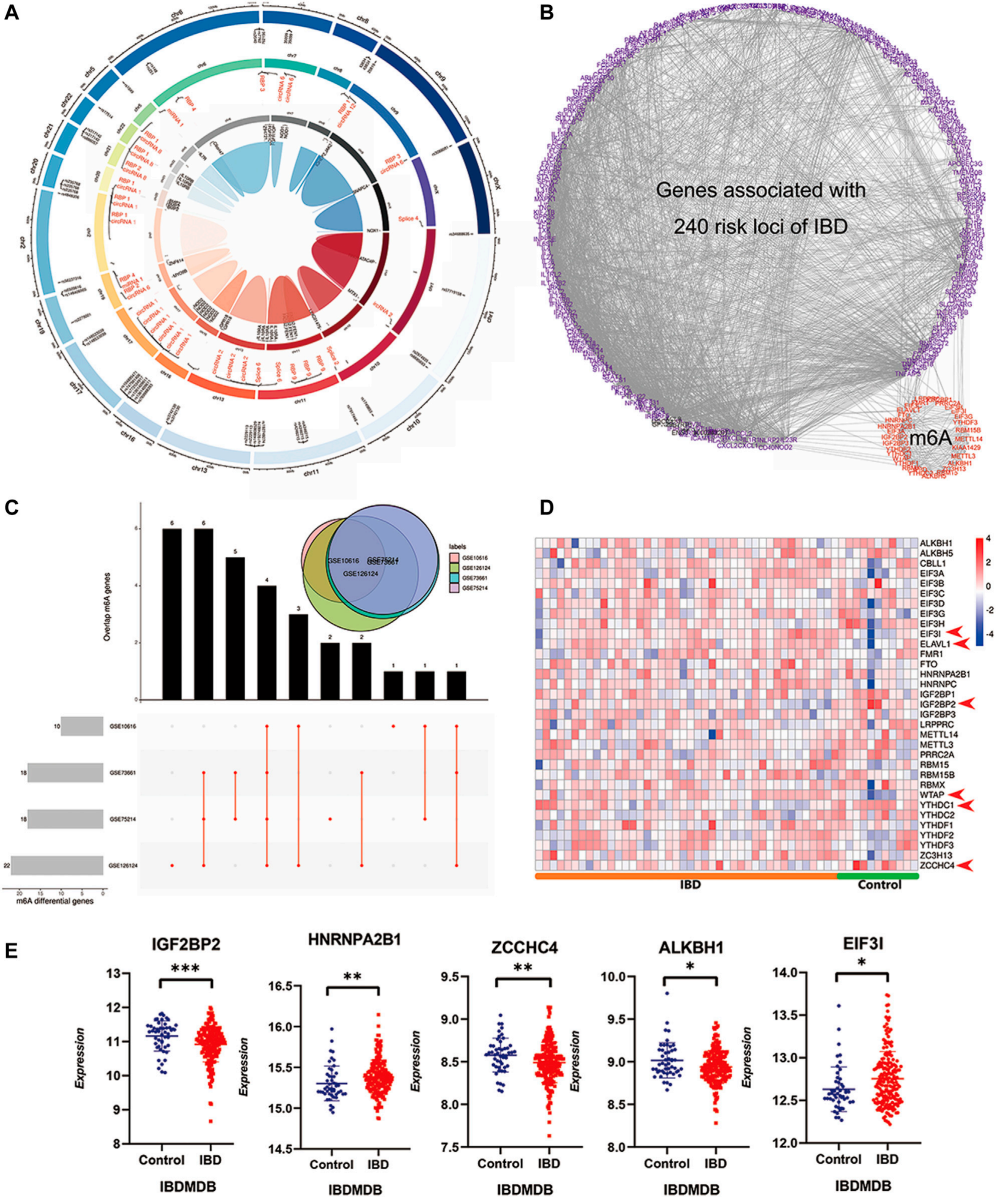
A widespread interaction and participation of m6A modifications in IBD. **(A)** is the Circos diagram of known m6A modifications in IBD associated risk SNPs based m6A sequencing. The outer circle indicates the positions of m6A modified IBD associated risk SNPs in chromosome, the middle circle is transcriptome regulation of risk SNPs, the inner circle is the risk gene name. **(B)** is the interaction network of 240 known IBD risk genes and m6A reader, writer and eraser genes. **(C)** is the UpSet diagram of differential m6A regulators in four independent discovery IBD cohorts. The upper right venn displays the overlaps of differential genes in four cohorts together. **(D)** is the heatmap of m6A reader, writer and eraser in validatory IBDMDB cohort. **(E)** is the expressing profile of top differential m6A regulators in the validatory IBDMDB cohort (t test, *p* < 0.05).

United Kingdom Biobank project analysis had identified 12.3 million variants that lead to changes in the encoded protein, and these variants were associated with human disease (Okada and Wang, 2021), and the IBD risk variants influenced the risk of IBD pathogenesis primarily as reported (Liu et al., 2015; de Lange et al., 2017). Moreover, m6A modifications usually read the specific SNPs as the signal label. To verify m6A’s role in IBD pathogenesis and pathophysiology, we explored the connection between the IBD risk variants and m6A modifications and the universal differential m6A regulators in IBD patients. Methylations related to IBD risk loci were identified based on public m6A-label-seq, miCLIP &DART-seq, or miCLIP data from RMVar (Luo et al., 2021). This analysis revealed that 49 IBD risk SNPs had methylation m6A sites. Circos analysis revealed these m6A modification sites’ chromosomal positions, transcriptome regulation, and risk genes (Figure 2A; Supplementary Table S1). An interaction network between 257 published IBD risk genes and m6A genes was used to elucidate high interactions (Figure 2B; Supplementary Table S1). Analysis of differential m6A gene expression between IBD patients and healthy controls using the aforementioned four discovery cohorts revealed 10, 18, 19, and 22 differentially expressed m6A genes in datasets GSE10616, GSE73661, GSE75214, and GSE126124, respectively (*p* = <0.05). The overlap map between these m6A genes in the discovery cohorts revealed universal and identical m6A methylation modifications in IBD patients (Figure 2C). Validation analysis using the IBDMDB cohort revealed expression differences between IBD patients and healthy controls (Figure 2D). Importantly, to achieve a better understanding of such changes, the expression profiles of partial m6A genes and differences were displayed in the IBDMDB validation cohort again (Figure 2E). We also enrolled twelve volunteers, including three Crohn’s patients, four ulcerative colitis patients, and five healthy controls. Their colonic expression of m6A regulators was measured by qPCR. Reader insulin-like growth factor 2 mRNA binding protein 2 (IGF2BP2) was downregulated in IBD patients compared to healthy controls (Supplementary Figure S1B, non-paired test, *p* < 0.05). Zinc finger CCHC-type containing 4 (ZCCHC4) was also downregulated, although no significance exists (Supplementary Figure S1B). Heterogeneous nuclear ribonucleoprotein A2/B1 (HNRNPA2B1) reached significance, but the tendency was not consistent (Supplementary Figure S1B, non-paired test, *p* < 0.05).

### An Insight on m6A Landscape in IBD

**FIGURE 3:**
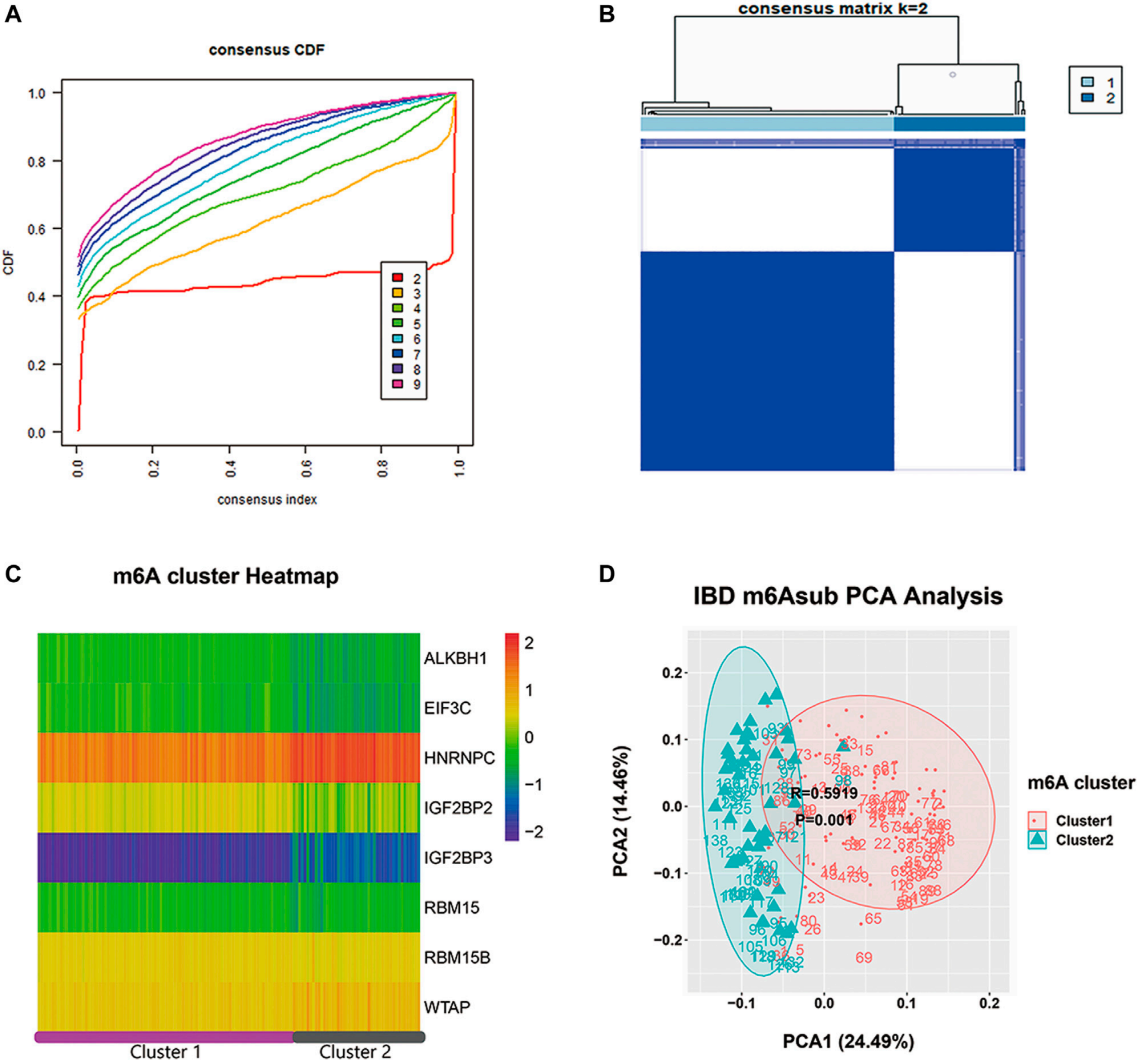
The consensus clustering analysis of m6A regulators in IBDMDB cohort. **(A)** is the consensus CDF diagram which show a good clustering is obtained when k = 2. **(B)** is the different clusters after consensus clustering by k = 2, **(C)** is the heatmap of m6A reader, writer and eraser expression in different clusters. **(D)** is the PCA analysis that shows well discrimination between two clusters.

**FIGURE 4:**
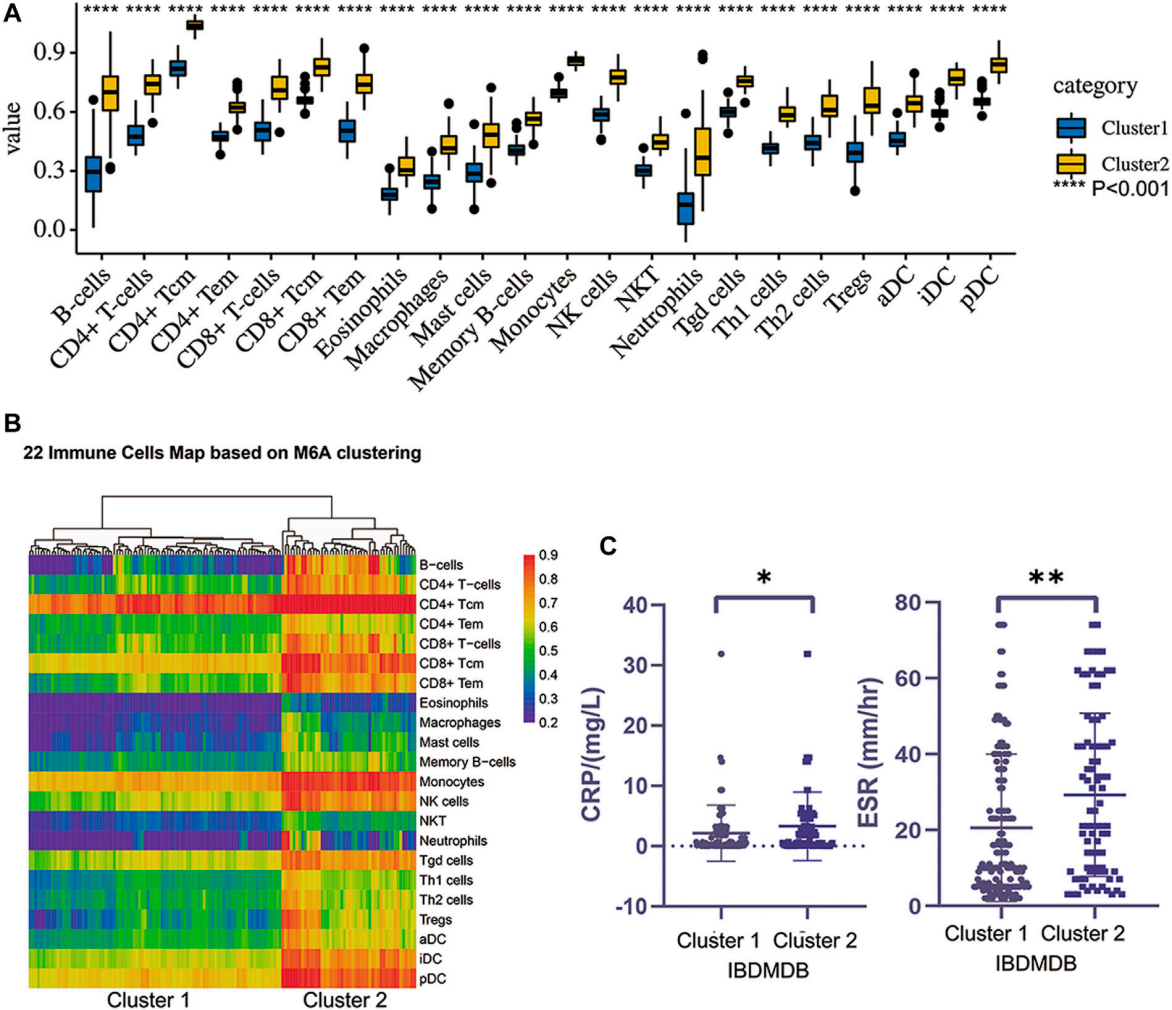
The different immune landscape and clinical phenotypes of two clusters obtained by consensus clustering. **(A)** is the different immune scoring of major 22 immune cells in two clusters (*p* < 0.01). **(B)** is the heatmap of the two clusters’ immune landscape. **(C)** is the difference of two clusters clinical indexes such as ESR and CRP (t test, *p* < 0.05).

To carefully understand the divergent roles of m6A modification in the IBD pathophysiology, we investigated m6A regulators’ sub-phenotypes among IBD patients. m6A modification is a reversible biological process, and different groups of m6A genes have opposite effects on the fate of RNA. Consensus clustering was conducted based on the IBDMDB m6A matrix to explore m6A subgroups among IBD patients. The findings showed clusters of IBD patients’ samples (*n* = 198), which appeared as “independent islands” in the map with K = 2, indicating that sub-clusters of samples exhibited common genomic m6A features (Figures 3A,B; Supplementary Table S2). In addition, the m6A expression profile of the sub-clusters was expressed as a heatmap, and PCA showed a distinction between two m6A clusters (Figures 3C,D; Supplementary Table S2). Dysregulation in the immune system plays an important role in IBD. Therefore, the immune scores of the different clusters were obtained from the Xcell website for 22 immune cell types for further analysis (Aran et al., 2017). Different m6A clusters displayed significant immune distance from each other, and the different immune phenotypes indicated different clinical features (Figures 4A,B; Supplementary Table S2). CRP and ESR are common clinical inflammation indexes for disease diagnosis and management. Moreover, the CRP and ESR data of these samples were retrieved from the IBDMDB database to further explore the clinical features of m6A clusters, and the analysis showed a significant difference (non-paired t-test; *p* < 0.05) between the two m6A clusters (Figure 4C; Supplementary Table S2). The sub-m6A-phenotypes’ different clinical features will guide to better disease diagnosis and management.

### Immune-Related m6A Methylation Landscape in IBD

**FIGURE 5:**
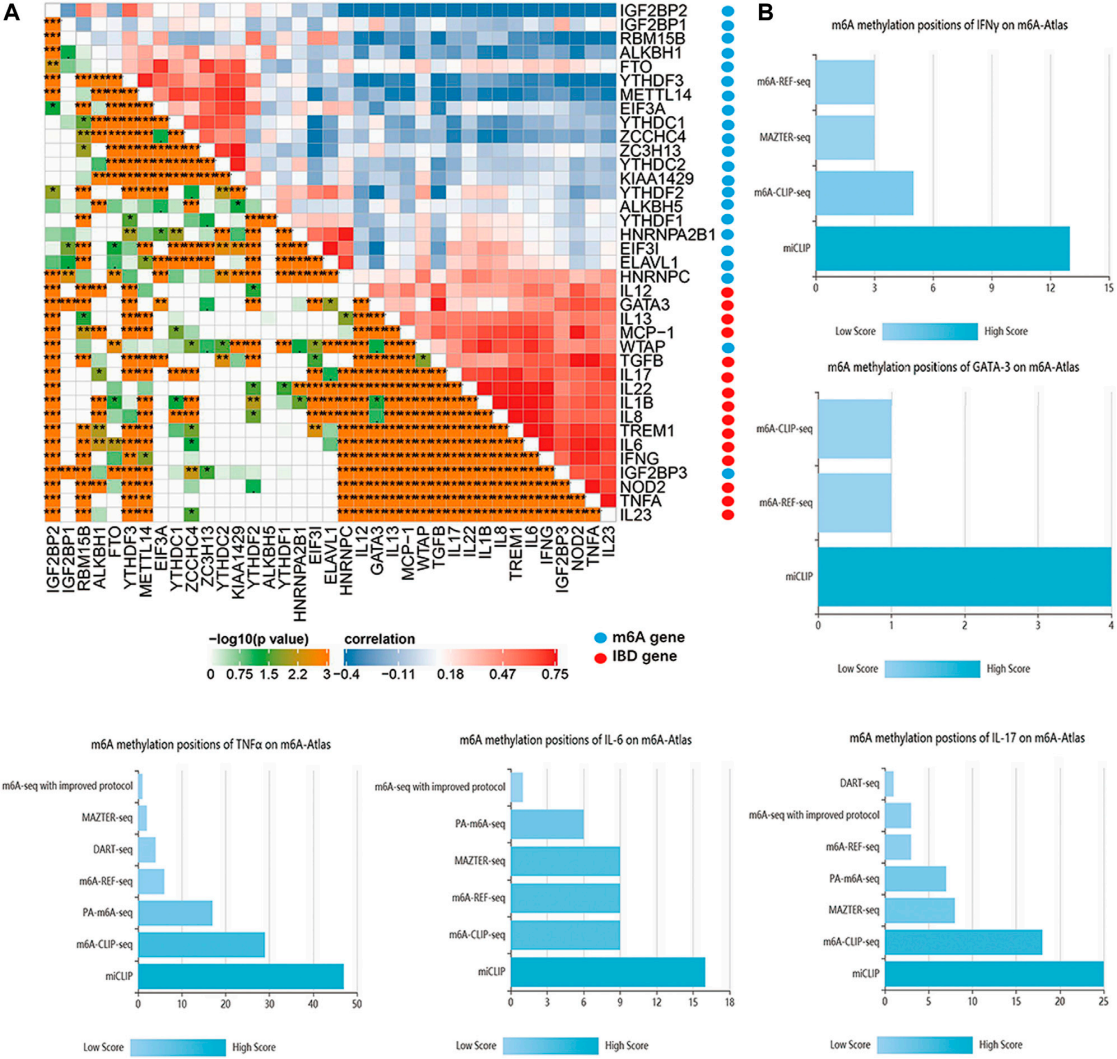
The interaction of m6A regulators and IBD core cytokines. **(A)** is the correlation map of m6A regulators and IBD core cytokines. **(B)** is the m6A methylation positions of IBD core cytokines based on different m6A detecting technology.

Sub-m6A-phenotypes have different clinical features. With no doubt, the pathophysiology of IBD exhibits typical immune dysfunctions (Chang, 2020), and m6A modifications are known to influence immune cells a lot (Winkler et al., 2019; Shulman and Stern-Ginossar, 2020). Therefore, it is natural that we further explore the immune-related m6A methylation landscape in IBD. Immune cytokines play an important role in the pathogenesis and outcome of IBD. For example, IL2, IL4, GATA3, IL9, IL13, TGBβ, and TNFα are implicated in UC (Ungaro et al., 2017), whereas IL4, IL6, IL12, IL17, IL21, IL22, IL23, TNF, and IFNγ are involved in CD (Roda et al., 2020). Different m6A clusters exhibit differences in immune profiles. Therefore, correlation analysis was performed between IBD core cytokines and m6A genes in the IBDMDB. The findings showed significant correlations among m6A genes and immune cytokines (Figure 5A). Furthermore, m6A modification characteristics of these cytokines were obtained from the m6A-Atlas database (Tang et al., 2021). The N6-adenosine methylation sites on these cytokines were explored using high-quality sequencing analysis (such as miCLIP, m6A-CLIP-seq, m6A-REF-seq, MAZTER-seq, and PA-m6A-seq). Details on m6A modifications of these immune cytokines are presented in Figures 5B–F; Supplementary Table S2.

### Role of m6A Modifications in Different Phenotypes of IBD

**FIGURE 6:**
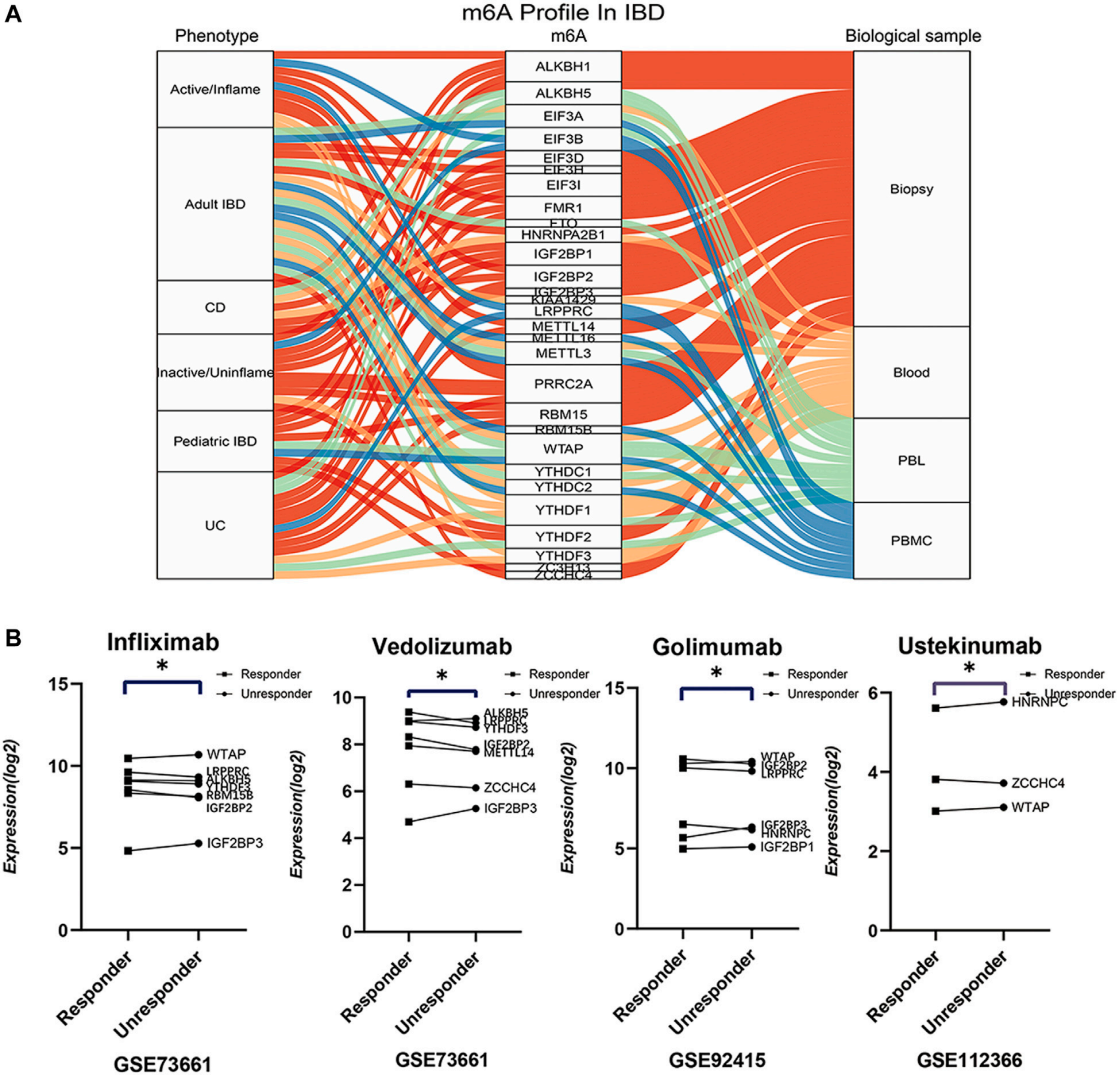
The globally differential expressions of m6A readers, writers and erasers in different phenotypes, sample sources, patient groups, and therapeutic response subgroups. **(A)** is the sankey diagram of differential m6A regulators between different phenotypes, sample sources, patient groups. **(B)** is the top differential m6A regulators between responder and unresponder by biologics such as infliximab, vedolizumab, golimumab, and ustekinumab.

Except for the pathogenesis and pathophysiology, the impressive m6A-IBD interactions reinforce us to further explore the effect of m6A modifications on clinical phenotypes and treatment of IBD. Global comparisons were carried out between UC and CD (GSE75214), inflamed and uninflamed tissues (GSE75214), adult IBD (GSE75214), pediatric IBD (GSE6989), IBD whole blood sample (GSE119600), IBD PBLs sample (GSE33943), and IBD PBMCs sample (GSE3365). The top five differentially expressed genes were obtained with *p* < 0.05. A Sankey diagram was generated to present the universal different m6A signatures among different clinical phenotypes (Figure 6A; Supplementary Table S3). In addition, m6A signatures were explored among different biological therapeutic response subgroups, and the findings showed differential expression of m6A genes among different response subgroups (Figure 6B; Supplementary Table S4). These findings indicate that m6A modifications have a significant effect on different IBD phenotypes.

## Discussion

Previous studies have not explored the integral role of m6A in IBD. For the first time, the current study explored a comprehensive landscape of m6A modifications in IBD. Analysis of the mechanism of m6A modifications shows that the multi-component m6A MTC (or “Writer”) catalyzes adenosine methylation by binding S-adenosylmethionine. METTL3 and METTL14 combine to form a complex core methyltransferase domain. Notably, METTL16 functions without forming a complex, and targets U6 snRNA and MAT2A mRNA, which is independent of the m6A deposition DRACH motif (Meyer and Jaffrey, 2017; Zaccara et al., 2019; Huang et al., 2020). Other regulatory subunits, such as WTAP, RBM15/RBM15B, KIAA1429 (VIRMA) and ZC3H13, play roles in anchoring the “Writer” in regions adjacent to mRNAs’ m6A sites. The common consensus m6A deposition motif is the DRACH motif (D = G, A, or U; R = G or A; and H = C, A, or U). MTC adds methyl to the adenosine within the DRACH motif (Linder et al., 2015). m6A is a reversible biological process modulated by several RNA demethylases (the “erasers”). For instance, FTO and ALKBH5 trigger demethylation of methylated adenosine (Frayling et al., 2007; Zaccara et al., 2019). Therefore, m6A modification is a dynamic RNA modification process involving m6A writers and erasers. Therefore, several RNA-binding proteins (“readers”) can identify the m6A signal in mRNAs and modulate mRNA fate by affecting splicing, translocation, decay, stabilization, and translation processes (Zaccara et al., 2019). m6A has been reported its potential connections with IBD, METTL3 and METTL14 deficiency in immune cell induce colitis; m6A eraser FTO protects IBD patients from adverse reactions after thiopurine treatment (Xu et al., 2021). The current research on m6A and IBD is scarce, so the cognition of m6A on IBD is limited and frustratingly (Xu et al., 2021). Therefore, there is a strong need for relevant exploration to provide more possibilities for the pathogenesis, clinical diagnosis, and treatment applications in IBD (Xu et al., 2021; Zhang et al., 2021). A better understanding of primary RNA modification in IBD will undoubtedly provide a new direction for the occurrence, development, and clinical intervention of IBD.

In order to understand the role of m6A modification in the pathogenesis of IBD, our results show IBD-associated variants could be modified by m6A and their associated gene expression potential changed. Disease-associated SNPs affect disease state, and several IBD-associated SNPs have been detected by GWAS and other techniques (Liu et al., 2015; de Lange et al., 2017; Huang et al., 2017). One example of this mechanism is the NOD2 gene, mutations that generate a non-functional version of NOD2 are a risk factor for Crohn’s disease, and NOD2 risk variants are associated with activated immune cells and fibrosis. Coding and noncoding variants could both influence the specific disease risk (Backman et al., 2021; Nasser et al., 2021). Correlations analysis of SNPs using m6A-seq showed an enrichment of SNPs in m6A-containing regions (Liu J.e. et al., 2020; Luo et al., 2021). Consequently, disease risk variants may modulate RNA fate and gene expression by RNA modification sensing. To explore the internal complex mechanism of IBD variants and their related genes, the current study performed bioinformatics analysis to offer a further insight on mechanisms of IBD pathogenesis. Out of the 232 IBD risk loci identified through GWAS, 122 were associated with a total of 157 genes (Furey et al., 2019). Genotypic variation can contribute to gene expression differences across individuals. Expression quantitative trait loci (eQTLs) are genomic regions with specific genetic variants, including SNPs, that are associated with levels of expression of one or more genes. Analysis of eQTL shows that IBD variants may represent m6A modification loci, which exert effects on the fate and expression of the risk gene (Furey et al., 2019). Moreover, risk genes exhibit several interactions with m6A genes. The RMVar database provides important IBD-related SNPs that are frequently modified by m6A through a combination of m6A-seq data and disease associated SNPs data (Luo et al., 2021). In addition, our provided network between IBD risk genes and m6A genes gives a direct impression of the potential role of m6A in IBD. These findings indicate that m6A plays a significant role in the pathogenesis of IBD, which would help with early screening and prevention of the disease. The specific testing panel targeting risk variants’ m6A modifications might promote the prevention of IBD’s occurrence.

The discussion of global m6A regulators’ changes in IBD patients’ colonic tissues puts the IBD-associated RNA modifications into a new state. Analysis of multiple independent data series showed that they shared differentially expressed m6A regulators in IBD cohorts, which indicated common phenomena of alterations of m6A-related genes implicated in the pathogenesis of IBD. A significant role in IBD was confirmed for the shared differentially expressed m6A genes in the IBDMDB validation cohort. The top common differential m6A regulator, IGF2BP2 (also known as IMP2, a m6A Reader), is a direct mTOR substrate that participates in glucose, lipid, protein, and energy metabolism (Dai, 2020), which are key events in the pathogenesis of IBD (Lloyd-Price et al., 2019; Ding et al., 2020). Pre-mRNA with m6A modifications detected by IGF2BP2 can be prevented from degradation in the P-body. In addition, IGF2BP2 can promote the export of premature mRNAs to the cytoplasm. IGF2BP2 protein in IGF2BP family promotes stability, inhibits decay and promotes storage of their target transcripts in a m6A-dependent manner, thus affecting mRNA fate and gene expression (Huang et al., 2018). The altered IGF2BP2 may modulate the stability, degradation, and storage of several important IBD genes, thus affecting IBD pathology. In addition, HNRNPA2B1 (Reader), another common m6A gene in IBD cohorts, promotes m6A modification and nucleocytoplasmic trafficking, thus facilitating effective production of interferons mediated by cyclic GMP-AMP synthase (cGAS)-STING (Wang L. et al., 2019). Dysbiosis of the gut virome is a common phenomenon in the process of IBD (Clooney et al., 2019). The cGAS-STING system is a vital virus-immune signaling pathway in which the gut virome dysbiosis deteriorates the impaired IBD innate immune system through cGAS-STING associated m6A modifications (Zheng et al., 2017; Wang L. et al., 2019). In addition to m6A readers, common IBD differential m6A writer ZCCHC4 plays an important role in methylating 28S rRNA, thus promoting ribosome assembly and translation, which in turn affects cell proliferation and growth (Ren et al., 2019). ZCCHC4 determines the fate of IBD core cytokines. Furthermore, m6A in the 5′UTR is small and is recognized by a multi-subunit interface of eIF3 involving eIF3a, eIF3I and other subunits. eIF3 recruitment to mRNA is a general mechanism for promoting translational (Wang et al., 2013; Choe et al., 2018). The eIF3 complex can modulate several important IBD pathophysiological events. New evidence is emerging that m6A reader, ELAVL1, could directly interact with Atg16l1 mRNA via its 3′ untranslated region and enhance ATG16L1 translation without affecting Atg16l1 mRNA stability (Li et al., 2020). Intestinal mucosa from patients with IBD exhibited reduced levels of both ELAVL1 and ATG16L1 (Li et al., 2020), and ATG16L1 is a crucial autophagy-related gene in IBD (Murthy et al., 2014). Importantly, we validated the bioinformatic discoveries in our IBD colonic tissues. Therefore, the m6A regulators’ dysfunction plays an unknown role in IBD, which needs to be explored further.

Immune dysfunction sustains an essential role in IBD. Immune cells such as T cells, macrophages, and dendrite cells exhibit differences between the m6A subgroups of IBD patients. Our results indicate that m6A modification influences the immune phenotypes and clinical inflammatory state of IBD. Several studies have explored the role of m6A modifications in the immune system (Han et al., 2019; Winkler et al., 2019; Shulman and Stern-Ginossar, 2020; Su et al., 2020; Wang et al., 2020). METTL3 is the core “writer” component of the MTC in m6A. A CD4-Cre loxP-flanked-METTL3 (METTL3^fl/fl^) mouse model exhibited more circulating naive T lymphocytes and less abundant activated CD4^+^ T cells, thus exerting a preventive role in the evolving colitis (Li et al., 2017). Moreover, T cells without METTL14, a m6A “writer” component of the MTC, showed similar phenomena (Lu et al., 2020). METTL3 and METTL4-deficient T helper cells do not induce colitis as they cannot differentiate into pathogenic effector T cells (Li et al., 2017). In addition to m6A writers, the m6A eraser ALKBH5 can modulate the naïve CD4^+^T cells’ infiltration and enhance the responses of CD4^+^ T cells (Zhou et al., 2021). These studies indicate the critical roles of m6A genes in T-cell homeostatic proliferation and differentiation (Li et al., 2017). Mice with a specific METTL3 knockout from Tregs displayed a systematic loss of the suppressive function of Tregs and could not perform m6A RNA modification (Tong et al., 2018). m6A modifications mediate Tregs function by IL-2-STAT5 signaling pathway (Tong et al., 2018). A recent study reported that CD4-Cre METTL14^fl/fl^ mice developed colitis, which was characterized by increased Th1 and Th17 cytokines and dysfunctional Tregs (Lu et al., 2020). Notably, a microbiome modulated by antibiotics alleviated colitis, indicating a microbiota-immune interaction in the model (Lu et al., 2020). Microbiota-immune dysfunction plays a key role in the development of IBD, thus m6A modifications may play a role in IBD-associated microbiota-immune dysfunction. DCs are innate immune cells that can stimulate T cells and present antigens, which is also a critical player in onset of IBD (Chang, 2020). Lipopolysaccharide (LPS) stimulating condition increases proliferation of DCs proliferated and upregulates its m6A modifications. METTL3-mediated m6A modification increases translation of immune cytokines, DC activation and DC-based T-cell response (Wang H. et al., 2019). Furthermore, macrophages can exert an extensive inflammation-modulating effect under m6A modifications (Yu et al., 2019). In addition, LPS can enhance the level and function of m6A Writer METTL3 in macrophages, and overexpression of METTL3 significantly alleviates the LPS-induced inflammatory response in an NF-κB signaling-dependent manner (Wang J. et al., 2019). IBD is an autoimmune disorder and presents significant dysregulation of innate and adaptive immune responses (Chang, 2020). The clustering findings showed different m6A gene signatures displaying different immune features owing to the reversible biological process of m6A modification. Different m6A gene clusters exhibited the landscape and state of distant immune cells, such as T lymphocytes, DCs, and macrophages. The m6A-related immune signature indicates different clinical indicators and phenotypes. Therefore, these key immune cells (CD4^+^ T cells, Tregs, DCs and macrophages) may be implicated in occurrence and development of IBD, and m6A modification is involved in maintaining homeostasis and functions of these immune cells. However, the role of m6A modification in mediating T cells, DCs, and macrophages in the pathogenesis of IBD is not fully elucidated, and should be explored further. Our results support the m6A related immune dysfunction, which sets a basis of the m6A-induced effects in the pathophysiology of IBD. Further m6A-based prediction of prognosis will help with risk stratification and more precise management of patients.

In addition to immune cells, critical immune cytokines are the essential targets for m6A modifications. GATA3 and TNFα are important cytokines in the mucosa damaging and comorbidities of UC(Ungaro et al., 2017); whereas IL6, IL17, TNF, and IFNγ are deeply involved in the CD. Our results support the influence of critical cytokines by m6A modifications and their tight relationship with m6A regulators in IBD. Eraser ALKBH5 deficiency reduces levels of IFNβ and impairs the innate immune response (Liu et al., 2019). YTHDF3 suppresses interferon production by promoting FOXO3 translation (Zhang et al., 2019). HNRNPA2B1 promotes m6A modification and nucleocytoplasmic trafficking of cGAS-STING, a well-known virus sensing system (Wang L. et al., 2019). In addition, it facilitates effective induction of IFNα/β production mediated by cGAS, IFI16, and STING (Wang L. et al., 2019). The role of gut virome dysbiosis, a common feature in IBD, has not been fully elucidated (Clooney et al., 2019; Liang et al., 2020). The findings of the current study showed that HNRNPA2B1 was a common differential gene among IBD microarray data. Therefore, an impaired IBD innate immune and gut virome dysbiosis may play a role through an m6A-associated viral sensing signaling. Inflammatory cytokines such as GATA3 are modified by m6A for degradation by KIAA1429 (Lan et al., 2019). IL6 mRNA is demethylated by m6A eraser ALKBH5 to inhibit translocation from the nucleus, and the production of IL6 is suppressed by m6A modification (Zhao et al., 2020). Moreover, apoptosis involved in the pathogenesis of IBD may affect immune and gut barrier function (Pedersen et al., 2014; Lin et al., 2017). Notably, apoptosis can be significantly modulated by m6A modifications (Vu et al., 2017; He et al., 2019). For example, m6A Writer METTL3 inhibits apoptosis (Vu et al., 2017; Cai et al., 2018), whereas its Eraser FTO and Reader YTHDF2 promote apoptosis (Huang et al., 2019; Zhong et al., 2019). Findings from the current study and previous studies indicate an extensive regulatory effect of m6A, not limited to apoptosis and immunity. Therefore, future IBD study design and therapy target development could focus on the m6A modifications.

Different phenotypes and therapeutic responses rely on genetic and environmental factors. m6A provides fundamental explanations of the different phenotypes such as flame and inflamed tissues, pediatric and adult patients; inconsistent biological differences including blood, PBLs, and PBMCs. m6A modifications induce genomic differential expression and distinct biological effects. Further studies on the m6A modification mechanism will further elucidate the pathophysiology of IBD. Another major concern is the clinical management of IBD, therapeutic agents should be well distributed to patients based on their different conditions. m6A accounts for an important part of biological response backgrounds of IBD patients. m6A modification provides a definition of the important distinctions between different biological response subgroups and offers a valuable basis for disease management.

As a primary mRNA modification, m6A contributes to a large group of disorders such as cancers, metabolism, immune, and others. Consequently, the progress of m6A’s effect on IBD would be a breakthrough in understanding the disease. We want to contribute to the m6A modification’s understanding of IBD’s pathogenesis, pathophysiology, and clinical phenotypes. However, our analysis has several shortages, such as limited clinical sample validation, lack of detailed exploration, and more. Due to the COVID-19 epidemic, critical lockdown, and restricted admission, recruiting volunteers is complex, and colonic biopsy is usually limited. Further IBD-related m6A details need to be uncovered.

In conclusion, the current study performed comprehensive analysis on the significance of m6A in IBD. The findings showed a global m6A gene difference, m6A associated SNPs, m6A clusters and different clusters’ immune signatures, and m6A features of different clinical phenotypes in IBD. The current study presents the IBD m6A modification network, including important immune cells, cytokines, and SNPs. This landscape provides information on the role of m6A modification in the progression of IBD. However, the specific m6A genes and corresponding modification mechanisms should be explored further, and their roles in IBD should be elucidated.

## Data Availability

Publicly available datasets were analyzed in this study. This data can be found here: The recruited microarray date including GSE10616, GSE73661, GSE75214, GSE126124, GSE6989, GSE119600, GSE33943, GSE3365, GSE73661, GSE73661, GSE92415, and GSE112366 are available at the GEO database (https://www.ncbi.nlm.nih.gov/geo/). The validatory IBDMDB data and relevant participants’ information are available at the IBDMDB database (http://ibdmdb.org). The functional variants involved in RNA modification are obtained from the RMVar database (http://rmvar.renlab.org). Data from m6A methylation sequencing is available at the m6A-Atlas (www.xjtlu.edu.cn/biologicalsciences/atlas).

## Supplementary Material

Supplementary Figure 1
**(A)** is the data processing procedure. **(B)** is the qPCR measure of some common m6A regulators in colonic tissues from three Crohn’s patients, four ulcerative colitis patients, and five healthy controls (non-paired t-test, **p* < 0.05, ns *p* > 0.05).
