# Supplementary figures and images for: A Broad m6A Modification Landscape in Inflammatory Bowel Disease
Source: Front Cell Dev Biol. 2022 Jan 19;9:782636. doi: 10.3389/fcell.2021.782636 (PMC8809481; doi:10.3389/fcell.2021.782636)

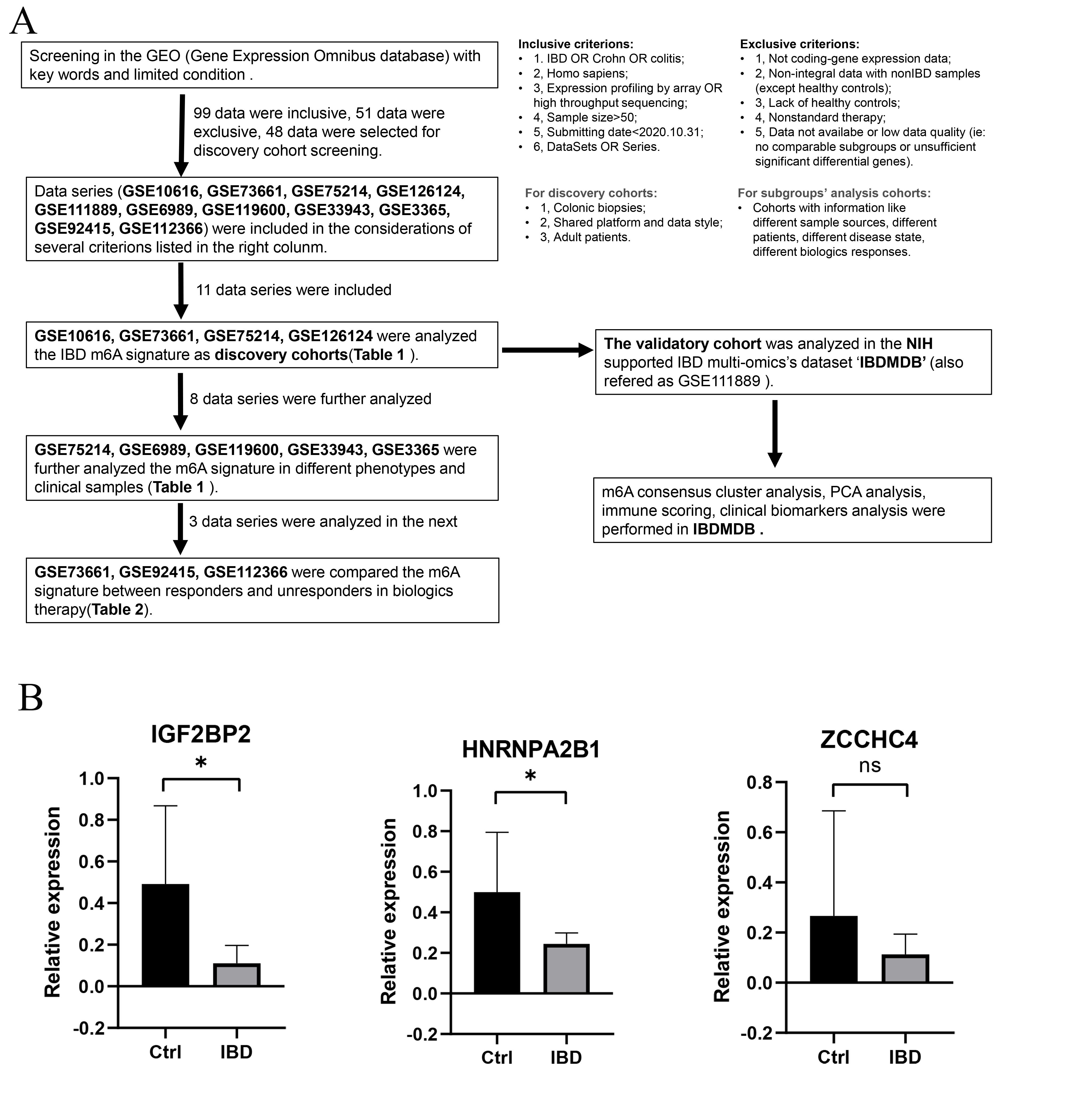

Supplement: Supplementary file 3 [file Image1.tif]
